# Evaluación del cumplimiento de los lineamientos en los menores de cinco años con desnutrición aguda

**DOI:** 10.15446/rsap.V25n2.104198

**Published:** 2023-03-01

**Authors:** Lurky E. Cadavid-Velásquez

**Affiliations:** 1 LC: Enf. M. Sc. Salud Pública. Universidad de Córdoba. Córdoba, Colombia lurkycadavidv@correo.unicordoba.edu.co Universidad de Córdoba Salud Pública Universidad de Córdoba Córdoba Colombia lurkycadavidv@correo.unicordoba.edu.co

**Keywords:** Desnutrición, calidad de la atención en salud, políticas de salud, evaluación de procesos, atención de salud, vigilancia en salud pública *(fuente: DeCS, BIREME)*, Protein-energy malnutrition, quality indicators, health care, process assessment, health care, public health surveillance *(source: MeSH, NLM)*

## Abstract

**Objetivo:**

Evaluar el cumplimiento de los lineamientos para la desnutrición aguda, moderada y severa en menores de cinco años del Departamento de Córdoba (Colombia).

**Materiales y Métodos:**

Se realizó un estudio descriptivo correlacional de corte transversal. Participaron 23 Unidades Primarias Generadoras de Datos (UPGD) de instituciones públicas que diligenciaron una encuesta física de acuerdo con los lineamientos de la normativa vigente, con un nivel de confianza de 95% y un máximo error permisible de 5%.

**Resultados:**

El 83% de las UPGD públicas no realiza búsquedas activas institucionales. El 74% no realiza mantenimiento de los equipos de medidas antropométricas. El 78% no garantiza la prescripción de la Fórmula Terapéutica Lista para el Consumo (FTLC). El 83% no presta la atención en salud con calidad. El 78% de los profesionales no está adherido al lineamiento técnico.

**Conclusiones:**

Los resultados son concluyentes, no hay adherencia e implementación de los lineamientos en las UPGD públicas y esto se refleja en la atención y los casos notificados al Sistema de Vigilancia Epidemiológica. Algunos reactivos se cumplen en su totalidad, pero estos no se reflejan en la atención integral.

## Abstract (en)

**Objective:**

To evaluate compliance with the guidelines for the management of acute, moderate, and severe malnutrition in children under five years of age in the department of Córdoba (Colombia).

**Materials and Methods:**

A cross-sectional correlational descriptive study was conducted with 23 Primary Data Generating Units (UPGD) from public institutions participating. The study involved completing a physical survey in accordance with the guidelines of the current standard, with a confidence level of 95% and a maximum permissible error of 5%

**Results:**

83% of the public UPGD do not carry out active institutional searches.74% do not perform maintenance on anthropometric measurement equipment. 78% do not guarantee the prescription of the therapeutic formula ready for consumption. Additionally, 83% do not provide quality healthcare, and 78% of professionals do not adhere to the technical guideline.

**Conclusions:**

The results are conclusive: there is a lack of adherence to and implementation of guidelines in public UPGD, and this is reflected in the quality of care provided and in the cases reported to the Epidemiological Surveillance System. While some criteria are fully met, they do not contribute to comprehensive care.

Existen muchos problemas de malnutrición alrededor del mundo, de los cuales la desnutrición es uno de los más graves, pues produce retraso del crecimiento y carencias de nutrientes y micronutrientes. La política de cada país es responsable de esta situación, pues permite la transformación de los entornos y la destrucción del medio ambiente, lo cual acelera el cambio climático y, por ende, produce inseguridad alimentaria. Por lo tanto, los niños con bajo peso al nacer presentan un alto riesgo de desnutrición 1,2.

El informe titulado "El estado de la seguridad alimentaria y la nutrición en el mundo", plantea la posibilidad de que los grupos poblacionales más vulnerables deterioren aún más su estado nutricional a causa de las repercusiones económicas causadas por el COVID-19 3.

Para el 2025, los Objetivos de Desarrollo Sostenible (ODS) fijaron la meta de reducir al 5% los niños menores de cinco años con emaciación en el mundo, pues, para 2020, 45,4 millones de niños menores de cinco años (6,7%) padecían emaciación 2,3. Es importante destacar que aún existen marcadas desigualdades en muchos países, las cuales establecen el estado de salud de cada individuo y repercuten en los estilos de vida, el comportamiento de la población, las condiciones de vida, las condiciones ambientales, las condiciones climáticas, las circunstancias psicosociales y los factores biológicos.

Las múltiples formas de malnutrición son evidentes en Colombia, principalmente por la inseguridad alimentaria que se refleja en los niños afectados por bajo peso para su talla y que presentan un mayor riesgo de muerte 3. Por lo tanto, la desnutrición como enfermedad de origen social es el resultado de la inseguridad alimentaria y nutricional y afecta principalmente a menores de cinco años.

La situación de Colombia influye principalmente en los determinantes sociales, que varían dependiendo de las manifestaciones políticas, económicas, sociales y culturales de cada región y además contribuyen a los problemas de salud pública. Para el año 2020, se notificaron al Sistema de Vigilancia en Salud Pública (SIVIGILA) 10.744 casos con desnutrición. Sin embargo, es importante aclarar que la pandemia por COVID-19 trajo consigo una disminución en la asistencia a servicios de salud, por lo cual disminuyó la notificación del evento 4.

El departamento de Córdoba, escenario de esta investigación, se ubica en la región del Caribe colombiano. Allí, el SIVIGILA reporta una prevalencia de 1,7% de desnutrición aguda, la cual es elevada teniendo en cuenta que la prevalencia nacional es de 1,6%. Dicho brevemente, esto indica que la prevalencia departamental está por encima de la nacional. En 2021, el Instituto Nacional de Salud (INS) reportó 560 casos y en 2022, a la semana epidemiológica 28, se han notificado 307 casos. Los resultados para 2022 pueden triplicarse por las inasistencias a los servicios de salud.

Según SIVIGILA, en los últimos años el departamento ha reportado un incremento en los casos de desnutrición, dato que se relaciona con los determinantes sociales de la salud de la Organización Panamericana de la Salud (OPS/OMS), los cuales identificaron ciertas necesidades visibles como: calidad de la vivienda, factores de tensión, condiciones de vida, relaciones estresantes, falta de apoyo de redes sociales, formas de nutrición, inactividad física, consumo de sustancias psicoactivas, respeto entre los diversos grupos, exposición y vulnerabilidad a los factores de riesgo, acceso a los servicios y programas de salud, pobreza en zonas rurales, poca orientación en cuidados nutricionales de la mujer durante y después del parto, falta de servicios de alcantarillado y agua potable, deficiencia en la calidad de la educación pública, violencia y trauma, desempleo, informalidad, inequidad, desigualdad, desequilibrio en el plato saludable y presencia de enfermedades prevalentes.

De Onis 5 hizo parte del estudio de medidas somáticas conformado por un comité de expertos para reevaluar el uso de la antropometría. Estos argumentaron que la anamnesis, la exploración física y los índices estáticos usados para comparar las dimensiones del niño son muy ventajosos porque no son invasivos, son fáciles de implementar para la recolección e interpretación de los datos, tienen bajos precios y se pueden aplicar universalmente para evaluar la proporción, el tamaño y la composición del cuerpo humano. Además, es posible valorar la evolución del estado de salud y nutrición mediante el seguimiento de intervalos regulares de los cambios que se van produciendo a lo largo del tiempo.

De acuerdo con lo anterior, la evaluación antropométrica del estado nutricional individual de un niño debe ser interpretada en el contexto de una evaluación comprensiva del estado de salud y enfermedad. También deben valorarse las condiciones genéticas y las enfermedades crónicas y hereditarias, pues si no se tienen en cuenta estos datos, el médico o pediatra puede dar un diagnóstico errado y no definir un criterio para el tratamiento médico integral 6.

Dentro de los propósitos que plantea la Resolución 2350 de 2020, destaca evitar prácticas nocivas, innecesarias e invasivas asociadas a muerte por desnutrición. Para realizar una buena valoración nutricional son indispensables las gráficas de medidas antropométricas de la Resolución 2465 del 2016 expedida por el Ministerio de Salud. Por estas razones, se adoptan los indicadores antropométricos y el protocolo de Vigilancia de Desnutrición Aguda, Moderada y Severa en menores de cinco años del Código 113, según el cual las UPGD tienen la responsabilidad de captar los casos, diligenciar la ficha de notificación y hacer las búsquedas activas institucionales.

Por la problemática presentada, es importante conocer la prestación de los servicios de salud en torno a la norma teniendo en cuenta que el departamento de Córdoba notifica considerablemente casos del evento de desnutrición aguda al SIVIGILA. Por lo tanto, se hace necesario evaluar que las entidades públicas cumplan con los criterios para brindar un servicio con calidad.

En relación con lo anterior, el estudio planteó como objetivo evaluar el cumplimiento de los lineamientos para la desnutrición aguda, moderada y severa en menores de cinco años del departamento de Córdoba (Colombia).

## MATERIALES Y MÉTODOS

Se desarrolló un estudio cuantitativo descriptivo correlacional de corte transversal durante el año 2022. Se contó con la participación de 23 UPGD públicas de los municipios del departamento de Córdoba.

Se diseñó una lista de chequeo de 28 reactivos con los lineamientos establecidos en las normas y los criterios epidemiológicos que deben ser utilizados en el proceso de vigilancia en salud con el fin de valorar su cumplimiento.

Los datos fueron obtenidos a través de la revisión de historias clínicas y evidencias de los lineamientos y protocolos implementados por cada entidad. La lista de chequeo incluyó una autorización por parte del secretario de salud departamental.

Para la tabulación de los resultados, el plan de análisis se realizó por medio de estadística descriptiva y se organizaron las preguntas y los datos por medio del programa Excel versión 2019. Posteriormente, se obtuvieron los porcentajes de cada ítem principal evaluado para obtener el porcentaje del cumplimiento del lineamiento en general. Una vez realizado esto, se hizo el análisis de cada uno de los reactivos.

## RESULTADOS

Caracterización demográfica de las UPGD públicas De las UPGD, 83,0% corresponden al primer nivel de atención y son de carácter municipal; 17,0% corresponden al segundo nivel de atención, son de carácter departamental y estás constituidas como Empresas Sociales del Estado (ESE). Las demás están organizadas como IPS autónomas.

La investigación considera que la desnutrición en el departamento de Córdoba disminuirá cuando las entidades prestadoras de los servicios de salud reconozcan y sean conscientes de que no hay adherencia al lineamiento de acuerdo con lo establecido por el Ministerio de Salud y Protección Social. Uno de los puntos más importantes en la valoración es que el 100% de las UPGD públicas de los municipios respondieron a los reactivos.

Gran parte de las UPGD de los municipios no notifican al código 113 del evento dentro de los términos establecidos. Algunas no realizan la captación a la ocurrencia de los casos a partir de las atenciones en menores de cinco años que cumplan con la definición operativa del caso. Por lo tanto, no realizan las búsquedas activas institucionales a través de los Registros Individuales de Prestación de Servicios de Salud (RIPS).

Al relacionar los tres reactivos, se encontró que la inoportunidad en la prestación del servicio es muy común, situación que retrasa la atención integral adecuada, en especial en los casos en que no se recibe atención por un mal diagnóstico. Esto evidencia el incumplimiento del lineamiento (Tabla 1).

**Tabla 1:** Notificación oportuna del evento y captación de la ocurrencia y búsquedas activas

Lineamiento	Sí cumple	%	No cumple	%
Notifican el evento en desnutrición aguda, moderada y severa en menores de cinco años en el municipio dentro de los términos establecidos	13	57	10	43
Captan la ocurrencia de los casos a partir de las atenciones en menores de cinco años que cumplan con la definición de caso	13	57	10	43
Realizan las búsquedas activas institucionales	4	17	19	83

Los análisis indican que este comportamiento se debe al desconocimiento del protocolo de atención integral a la desnutrición aguda, la falta de adherencia de los lineamientos o al desconocimiento de esta.

Se determinó que la mayoría de las UPGD no realizan mantenimiento de los equipos de medidas antropométricas. Asimismo, el periodo de los controles de estos y la empresa que los lleva a cabo no cumplen con los requisitos para tallar y pesar. Las entidades utilizan marcas avaladas por la OMS, pero los implementos son obsoletos o están deteriorados (tallímetro, infantómetro, balanza, cinta para perímetro braquial (Tabla 2).

**Tabla 2:** Mantenimiento de los equipos antropométricos y entidades que cumplen con los lineamientos de la OMS

Lineamiento	Sí cumple	%	No cumple	%
Realizan mantenimiento de los equipos de medidas antropométricas, cada cuanto lo hacen y que empresa lleva el control	6	26	17	74
Los equipos de medidas antropométricas cumplen con los requisitos para tallar y pesar, la marca es avalada por OMS (tallímetro, infantómetro, balanza, cinta para perímetro braquial)	9	39	14	61

Estos dos reactivos guardan una relación estrecha. Dependiendo de la funcionalidad de los equipos, se garantiza el diagnóstico y se definen los casos que corresponden al evento teniendo en cuenta la definición operativa del caso. En el momento de la verificación del lineamiento, se solicitó evidencia de los controles y la certificación para el funcionamiento de los equipos. Lo más significativo es que las entidades de los municipios donde se evidenció esta problemática tienen población con enfoque diferencial de población indígena. Por lo tanto, no se garantiza una atención integral con calidad.

En la Tabla 3, se describe el porcentaje que no garantiza o desconoce la prueba de apetito y la prescripción de la Fórmula Terapéutica Lista para el Consumo (FTLC).

**Tabla 3:** Prescripción de FTLC y la prueba de apetito

Lineamiento	Sí cumple	%	No cumple	%
Garantizan la prescripción de la FTLC a través de la herramienta MIPRES a los niños con desnutrición aguda moderada y severa de acuerdo con lo indicado en la Resolución 2350 del 17 de diciembre del 2020	5	22	18	78
Conocen el procedimiento para realizar la prueba de apetito y la edad en que se realiza la prueba	10	43	13	57

Con el fin de verificar la entrega del tratamiento FTLC, se buscó en las historias clínicas de los niños diagnosticados con desnutrición aguda la solicitud de la prescripción de productos de soporte nutricional mediante la herramienta tecnológica MIPRES 7.

Algunas entidades no están enroladas, y sin la gestión para dicho enrolamiento, algunos pediatras se resisten al cambio de tratamiento y a la vez lo desconocen.

## DISCUSIÓN

La notificación oportuna, la captación de los casos y las búsquedas activas institucionales son algunas de las problemáticas que debilitan el manejo del evento de desnutrición aguda en las UPGD.

El no mantenimiento de los equipos que no cumplen con los requisitos para tomar las medidas antropométricas es aún más preocupante, ya que no ayuda a definir el diagnóstico.

No garantizar la entrega de la FTLC, no estar enrolado a MIPRES y no conocer el procedimiento de la prueba de apetito, compromete aún más la vida del menor diagnosticado con desnutrición.

Estos hallazgos revelan que los elementos enmarcados en los resultados no garantizan la prestación de servicio con eficiencia, eficacia y calidad. Por lo tanto, la salud de los menores puede ser crítica, lo cual repercute en el fallecimiento del infante por la no definición y captación oportuna del diagnóstico ni la entrega del tratamiento indicado por la norma.

Las intervenciones de los profesionales (médico, enfermera, auxiliar de enfermería, nutricionista, psicólogo y trabajador social) reflejan un grave problema, ya que la remisión, intervención y seguimiento de los casos identificados no están recibiendo una atención integral e integrada. En el primer nivel de atención, deben ser valorados por un equipo interdisciplinario de primer nivel. Por lo tanto, todos los profesionales que hacen parte del equipo deben capacitarse periódicamente.

El seguimiento interdisciplinario a estos pacientes es del 39%, lo cual constituye el octavo reactivo con menor cumplimiento por parte de las UPGD públicas. Además, hay ausencia total del seguimiento en las áreas de trabajo social, nutrición y psicología.

Adicionalmente, el sistema político del departamento de Córdoba y de cada municipio impone barreras para la continuidad laboral de los profesionales, lo cual obstaculiza el proceso de atención con calidad.

Otras de las grandes barreras es el cambio anual de médicos que realizan las pasantías y el año rural, pues llegan al servicio sin experiencia en el proceso de atención y desconocen el protocolo, los lineamientos y las medidas antropométricas para brindar atención integral. Por esta razón, las universidades que tienen el programa de medicina, las direcciones locales de salud departamentales y municipales y las UPGD deben articularse para garantizar los procesos de atención con calidad.

Existen diversas investigaciones en las que se valora la prestación de los servicios de salud de los niños menores de cinco años, así como la prevalencia de desnutrición en una población determinada, sin embargo, son escasos los proyectos en los que se investiga si el cumplimiento de la norma se está llevando a cabo. Es posible que tales investigaciones se realicen al interior de cada institución para valorar la prestación de servicios, sin embargo, estos, por razones obvias, no son publicados.

El lineamiento para la atención integral a la desnutrición aguda constituye una política que se debe cumplir para contrarrestar la elevada prevalencia de las enfermedades en salud pública y mejorar la calidad de vida de los menores de cinco años que padecen la enfermedad. Es fundamental que se aplique en todas las UPGD, a pesar de esto, muchas entidades no cumplen y pasan por alto los procedimientos.

Esta situación puede indicar que en Colombia algunos centros de atención tanto públicos como privados no están cumpliendo a cabalidad con las normas técnicas propuestas por el Estado. Cabe destacar que el equipo de algunas entidades de salud no se preocupa por el fortalecimiento de capacidades, el cual se debe realizar periódicamente.

Es importante resaltar que en la investigación se encontró que un 57% de los cuidadores aseguran conocer los signos de alarma para una consulta oportuna por desnutrición, pero no actúan de forma inmediata. Esto quiere decir que los profesionales brindan educación a través de la ruta de promoción y mantenimiento establecida por la Resolución 3280 del 2018, pero esta información es vaga y carente de detalles sobre los riesgos reales para menores de cinco años. Por ello, muchos manifiestan no darle valor a la educación recibida. Algunas instituciones no tienen claridad en la activación de la Ruta de Atención Integral en Salud debido a la falta de compromiso en la implementación de esta y a pesar de que han recibido varias capacitaciones. Al igual, a algunos profesionales les falta calidad humana, comunicación, estabilidad emocional, empatía y atención al detalle para actuar ante la necesidad de los pacientes.

El 20% de los municipios del Departamento de Córdoba tienen activo el Comité de Seguridad Alimentaria y Nutricional (SAN). Este resultado indica que no hay programas o proyectos establecidos en los municipios para las familias y los niños notificados por el SIVIGILA.

Otro punto importante es que todas las UPGD tienen que estar enroladas en el aplicativo MIPRES para responder al tratamiento. En muchas ocasiones, estas manifiestan quejas porque los directivos no gestionan la inscripción al aplicativo y algunas veces las Empresas Administradoras de Planes de Beneficios (EAPB) afirman que hay desabastecimiento de la FTLC en el país.

Los resultados de la presente investigación son concluyentes y ponen en evidencia la necesidad de fortalecer las capacidades de todos los profesionales en salud. Las UPGD deben garantizar equipos de medidas antropométricas avalados por la OMS y los mantenimientos periódicos y las certificaciones anuales. Además, es indispensable fortalecer en los profesionales la adecuada identificación, tratamiento y seguimiento de los casos diagnosticados con desnutrición aguda implementando la Resolución 2350 del 2020 expedida por el Ministerio de Salud a través de la cual se adopta el lineamiento técnico para el manejo integral de atención a la desnutrición aguda moderada y severa en niños de 0 a 59 meses.

Además, es indispensable darles continuidad laboral a los profesionales en el área de trabajo en la que están capacitados y fortalecer periódicamente sus capacidades, especialmente cuando haya nuevas contrataciones de personal de salud.

Por todo lo anterior, es necesario realizar más investigaciones de este tipo en otros departamentos de Colombia para que el Estado exija la implementación y el fortalecimiento en los niveles de atención (DLS, UPGD, EAPB).

Estos aspectos integran el Plan Nacional de Seguridad Alimentaria y Nutricional 2012-2019 (PNSAN) y son una de las metas del Plan Decenal de Salud Pública 2022-2031 para reducir la mortalidad infantil por desnutrición.

Con respecto a las acciones de política pública a desarrollar en Colombia, los resultados del estudio fundamentan la relevancia de la valoración y atención integral a los menores de cinco años con desnutrición aguda moderada y severa. Esta afección tiene consecuencias negativas en la salud, los derechos de los niños y la economía de la población ♦
